# Neuropsychology in Multidisciplinary Stroke Care: Clinical Feasibility of the NINDS-CSN Vascular Cognitive Impairment Harmonization Standards

**DOI:** 10.1155/2014/216024

**Published:** 2014-07-20

**Authors:** Dong Y. Han, Amelia J. Anderson, Jana E. Jones, Bruce P. Hermann, Justin A. Sattin

**Affiliations:** ^1^Department of Neurology, University of Kentucky, 740 S. Limestone, Lexington, KY 40536, USA; ^2^Department of Neurology, School of Medicine and Public Health, University of Wisconsin, 1685 Highland Avenue, Madison, WI 53705, USA

## Abstract

As a significant number of stroke victims exhibit cognitive impairment, neuropsychological assessments can enhance poststroke management by identifying pertinent cognitive sequelae and providing salient care recommendations. However, due to operational differences between traditional neuropsychology and stroke services, neuropsychological assessments often remain underutilized in stroke care. We developed a novel care model that incorporated neuropsychological testing into a comprehensive stroke program using the modified vascular cognitive impairment (VCI) half-hour assessment protocol proposed by the National Institute of Neurological Disorders and Stroke—Canadian Stroke Network (NINDS-CSN). The test batteries were administered during the patients' acute admissions and then again upon follow-up in the multidisciplinary stroke clinic. Patient and provider satisfaction was then evaluated. Surveys revealed high provider satisfaction with improved clinic efficacy, improved data turnaround time, and with value neuropsychology services added to the comprehensive stroke program. Results from the 18-item industry standard Press-Ganey surveys showed all scores above 4.4/5.0 for patient satisfaction. This clinic garnered high provider and patient satisfaction after the first year. The (modified) NINDS-CSN VCI assessment protocol demonstrated clinical feasibility, suggestive of an efficient method of providing focused neuropsychological services in a clinical setting that otherwise prohibits traditional, comprehensive cognitive assessments.

## 1. Introduction

Stroke is one of the leading causes of long-term disability in the world, and in the United States, an average of one stroke occurs every 40 seconds. By this estimate, 795,000 new or recurrent strokes occur each year, with annual direct and indirect cost of cardiovascular disease and stroke in the United States being estimated at $312.6 billion [1]. In addition to the physical disabilities that often follow stroke, the risk factors that make individuals prone to stroke and cerebrovascular disease also place them at risk for potentially devastating cognitive impairments [2, 3]. Among stroke survivors, about 64% exhibit cognitive impairment [4] and up to a third develop dementia [5–7]. Research has demonstrated that these cognitive impairments are central to poststroke outcomes. Wagle and colleagues [8] found that estimates of cognitive functioning taken 2 to 3 weeks poststroke strongly predicted patients' practical functioning, measured by modified Rankin Scale scores, after 13 months of poststroke recovery. In addition, cognitive impairment measured 3 months after first-ever stroke has been associated with increased risks of death and disability 4 years later [9].

The cognitive impairments associated with stroke are heterogeneous [10], depending on the location and severity of individual ischemic lesions, preexisting microvascular lesions, and the patient's premorbid cognitive capacity, among other considerations [11]. Accordingly, there is significant within-group variability in even the best-established cognitive syndromes associated with stroke occurring in discrete vascular territories. In addition, a wide range of psychiatric symptoms can arise after stroke, related to both the ischemic lesions themselves and the significant psychosocial adjustments patients and their families often have to make after stroke [12]. These psychiatric comorbidities have a powerful influence over both quality of life [13] and functional outcomes. In a sample of 51,119 ischemic stroke patients, those patients diagnosed with depression or other mental health concerns had an increased risk of death 3 years poststroke, even after the influences of other chronic conditions were statistically controlled [14].

These points highlight the importance of proactively monitoring stroke patients' cognitive, psychiatric, and functional outcomes during outpatient follow-up. Indeed, a “holistic, comprehensive, interactive approach of an interdisciplinary team” has been regarded as the “hallmark of stroke rehabilitation” [15]. In such a model, domain-specific neuropsychological assessments are integral to supporting stroke patients' quality of life and increasing subsequent patient satisfaction with stroke-related healthcare services. Acknowledging this, recent harmonization standards called for routine, standardized cognitive and mood assessment in both clinical practice and research with stroke patients [16].

However, there is scant literature addressing the feasibility of multidisciplinary stroke care models incorporating cognitive, neurobehavioral, and neuropsychiatric outcomes. In the available literature, it is clear that (a) neuropsychological assessments remain underutilized in pertinent settings and (b) there is little standardization in the methodology of such assessments. In a recent review of stroke research conducted from January 2000 to October 2011, only 488 of the 8826 relevant studies (6%) included cognitive or mood assessment scales [17]. Within that 6%, assessment methods were highly variable, as researchers used 367 different assessment measures among these studies. This enormous variability renders comparing data between studies quite difficult.

Moreover, clinical services that hope to integrate neuropsychological assessment into poststroke care may find its practical implementation challenging. Given the time-sensitive nature of stroke, timely data transfer between providers is crucial not only in achieving the highest patient care standards, but also in promoting patient and provider satisfaction. However, due to operational incompatibilities between traditional neuropsychology clinics and stroke neurology services in most settings, routine neuropsychological assessments are rarely available in the acute poststroke period, and outpatient follow-up appointments are often substantially delayed. These factors often limit provision of neuropsychological assessment in poststroke care, and timely data transfer often remains an ideal rather than a practical, achievable standard.

At our tertiary care center, inpatient neuropsychological assessment services were not regularly available for stroke patients, and the outpatient referral system for neuropsychological testing was cumbersome. Patients' quantitative cognitive performance data from neuropsychological testing were seldom available at the time of their stroke clinic posthospitalization follow-up neurology appointments. These systemic barriers brought to our attention the need to implement a streamlined, interdisciplinary model of care that directly incorporated neuropsychology into standard poststroke care.

This project aimed to enhance patient services by implementing a multidisciplinary model of poststroke care that more closely integrated neuropsychology and neurology services. We anticipated that such a service would enhance acute poststroke care by (a) making inpatient neuropsychological services part of the standard of care. This would allow for tailored discharge recommendations based on efficient, systematic examination of patients' poststroke cognitive limitations. We also predicated that the benefit of the model would extend to outpatient follow-up practice by (b) providing quantitative measurements of poststroke cognitive recovery, (c) improving patient satisfaction with their poststroke care by mitigating logistical burdens, and (d) improving provider satisfaction with the neurology-neuropsychology collaboration by promoting direct communication and rapid data transfer between providers. We aimed to successfully develop and implement this care model within a 12-month timeframe, thus (e) providing preliminary data regarding the tolerability, feasibility, and utility of the neuropsychological assessment protocol for vascular cognitive impairment proposed by the international harmonization standards for poststroke care endorsed by the National Institute of Neurological Disorders and Stroke and the Canadian Stroke Network [16] (NINDS-CSN VCI protocol).

## 2. Materials and Methods

### 2.1. Multidisciplinary Stroke Clinic Implementation

The multidisciplinary stroke clinic was developed and implemented via collaboration between the neuropsychology and stroke neurology services within the Department of Neurology at the University of Wisconsin Hospital and Clinics (UWHC). Financial support for the multidisciplinary stroke service was provided both through the development of a postdoctoral fellowship in stroke neuropsychology, funded by UWHC, and provision of the Ambulatory Care Innovation Grant, an in-house quality improvement program funded by Physicians Plus Insurance Company and the University of Wisconsin Medical Foundation. Total cost for the project, including support of the stroke neuropsychology postdoctoral fellow and purchase of required materials, was under $100,000. After initial implementation, the neuropsychology section of the stroke multidisciplinary team became self-sustaining with service-based revenues.

#### 2.1.1. Inpatient Care

To incorporate neuropsychology into inpatient poststroke care, the stroke neuropsychology postdoctoral fellow was embedded within the inpatient stroke service's morning rounds on two mornings each week. Patients were identified as appropriate for neuropsychological testing on the basis of the fellow's clinical observations, in cooperation with the attending or resident neurologist, occupational therapist, speech-language pathologist, and nurses, who made rounds with the neurology team. Patients so identified were then administered the half-hour NINDS-CSN VCI neuropsychological assessment protocol. Results from this brief inpatient battery; accompanied by salient recommendations for patient supervision, return to school, work, and driving, and medicolegal decision-making capacity, among other considerations; were provided to the stroke team verbally and then in report form via the hospital's electronic medical record system. These recommendations were incorporated into the patient's discharge plan at the discretion of the attending stroke neurologist.

#### 2.1.2. Transitional Care

As part of the transition of care from discharge to outpatient follow-up, stroke patients were engaged in communication and treatment planning in two ways. First, patients were engaged in a three-way conference call between the inpatient unit clerk and the stroke program coordinator in order to schedule the multidisciplinary poststroke follow-up appointment, and this appointment was included in the inpatient stroke service discharge instructions. Further, informational resources, including letters, brochures, and instructions, were mailed to patients in order to again provide a brief description of the follow-up appointment, the nature of stroke, the purpose and nature of neuropsychological assessment, and resources for additional care.

#### 2.1.3. Outpatient Care

Upon discharge from the inpatient stroke service, an outpatient follow-up appointment was scheduled for each patient for 8 to 12 weeks from discharge during neurology clinic time designated for multidisciplinary stroke services. Each of these follow-up appointments was scheduled as a 2-hour block, with neuropsychological assessment scheduled 1 hour prior to the patient's follow-up appointment with the stroke neurologist. During the neuropsychology portion of the visit, a brief interval history was taken via clinical interview with the patient and any available family members or caregivers, and the NINDS-CSN VCI protocol was repeated in order to provide quantitative measurements of interval cognitive change. Scoring and preliminary interpretation of the test data were performed on-site in the clinic. Results were then provided immediately to the attending neurologist prior to the neurology appointment time, using a side-by-side graphical representation of Time 1 and Time 2 test results. The neurologist then incorporated the results of the neuropsychological testing in their counseling and treatment plan for the patient, often focusing on issues related to capacity for independent living and return to usual activities, such as work and driving.

### 2.2. Patient and Provider Satisfaction

After a pilot year, modified Press-Ganey scales, industry-standard 18-item surveys, were used to assess patient satisfaction with the multidisciplinary stroke clinic model. Specifically, the Press-Ganey scales assessed patient satisfaction with the clinic's appointment logistics, including the scheduling process, access to providers, and communication with care providers. In addition, a 10-item survey assessing stroke neurologists' satisfaction with the new multidisciplinary clinic was administered 12 months after implementation. This survey evaluated providers' satisfaction with clinic scheduling procedures both before and after implementation of the multidisciplinary clinic, data turnaround time both before and after clinic implementation, perceived value of the service to patients, perceived patient satisfaction with their care, and estimation of the clinic's added value to the comprehensive stroke program as a whole. Institutional review board was consulted, and deidentified data use was authorized given that the nature of the data assessed clinical quality improvement.

## 3. Results and Discussion

### 3.1. Inpatient Care

The first goal of the multidisciplinary stroke clinic project was to integrate neuropsychology into the standard of care for stroke patients in our care center. During the 12-month pilot implementation of the multidisciplinary stroke clinic, 114 patients were seen for inpatient cognitive evaluations.

### 3.2. Outpatient Care

Second, we hoped to extend the benefit of neuropsychology's integration to outpatient follow-up care by improving patient satisfaction with poststroke care, in part, by mitigating logistical burdens and improving provider satisfaction with the neurology-neuropsychology collaboration by promoting direct communication and rapid data transfer between providers.

#### 3.2.1. Appointment Utilization

Of the 114 stroke patients seen for inpatient cognitive evaluations, 67 patients returned for outpatient follow-up in the multidisciplinary stroke clinic. Remaining 47 patients initially seen were not followed up for multiple clinical reasons, for example, death, out of state residence, being cognitively normal deemed at initial evaluation prior to discharge, or being profoundly impaired with no clinical need for further evaluation. Provision of this service required 67 appointment slots in the multidisciplinary stroke clinic, rather than requiring 134 (67 × 2) separate time slots divided between the outpatient neuropsychology and stroke neurology services. This scheduling benefit required patients to travel to the hospital once for their follow-up appointments rather than making separate trips for neuropsychology and stroke neurology appointments. This represents significant mitigation of the logistical burden on patients, reducing travel costs and increasing feasibility for significantly disabled patients and their caregivers.

#### 3.2.2. Mean Wait Time

Prior to the implementation of the multidisciplinary stroke clinic, patients' mean wait time for a poststroke neuropsychological assessment was 5 to 10 months after their discharge from inpatient care. This delay was reduced from 1 to 3 months after implementation of the multidisciplinary stroke clinic, decreasing mean wait time by 7.26 months, a 78.32% decrease in patient waiting time. The 1 to 3 months wait time was also deliberately implemented to gauge poststroke cognitive recovery correlating with the first 90 days of anticipated poststroke improvement.

### 3.3. Patient and Provider Satisfaction

#### 3.3.1. Patient Satisfaction

Survey results indicated exceptionally high patient satisfaction with the multidisciplinary stroke clinic, as measured via the Press-Ganey scales. Composite responses to the Press-Ganey scales indicated exceptional patient satisfaction with all domains of service within the multidisciplinary stroke clinic, including logistics and scheduling, patient/provider interactions, patients' perceptions of care quality, and patient confidence in providers. As measured on a self-report Likert scale ranging from 0 to 5, with 0 representing the worst possible rating and 5 representing the best possible rating, patients rated their multidisciplinary stroke clinic care experience in all Press-Ganey assessed domains as at least a 4 out of 5 (range = 4.36 to 5.00). To review patient satisfaction ratings for all domains, see Table 1. Data are limited to those only collected during the project time frame and do not represent the total number of patients seen in clinic. Accordingly, at the time of data analysis, sample size was small (*N* = 16). This represented a 38% survey response rate.

#### 3.3.2. Provider Satisfaction

Responses to a 10-item survey of provider satisfaction indicated high provider satisfaction across domains assessed, including providers' perception of improved efficacy of clinic operations and interdepartmental data transfer time after implementation of the multidisciplinary stroke clinic model. Provider survey results also indicated high provider satisfaction with neuropsychology's added value to the comprehensive stroke program. Again, providers rated their satisfaction with the multidisciplinary stroke clinic care model in all assessed domains as at least a 4 out of 5 (range = 4.25 to 4.75). To review provider satisfaction ratings, see Table 2.

Stroke neurologists noted practical benefit from the incorporation of neuropsychology into post-troke services, based on enhanced triage capacities, efficient communication between providers, and rapid data transfer regarding patient outcomes. As neuropsychology was directly embedded into the comprehensive stroke service, and the stroke neuropsychology fellow was available to provide direct input into patient referrals, making it more likely to choose those patients who could best tolerate the NINDS-CSN VCI battery and whose neuropsychological data would likely augment clinical decision making. Effective communication was greatly facilitated, as providers had in-person contact during rounds two mornings a week and in multidisciplinary clinic one afternoon weekly. In addition, for the patients served during the 12-month pilot program, both inpatient and outpatient neuropsychological evaluation data were available during patients' acute stroke admissions and outpatient follow-up appointments. This allowed providers to make recommendations regarding return to school, work, driving, and other usual activities—at least in part—based on quantitative measurements of patients' cognitive capacities rather than relying on clinical judgment alone.

### 3.4. Basic Feasibility and Utility of the NINDS-CSN VCI Protocol

The stroke neuropsychology fellow and attending neuropsychologists commented favorably on the general clinical utility of the NINDS-CSN VCI half-hour protocol. First, the protocol could be completed in approximately one hour by most patients, including a focused clinical interview. In addition, the protocol was easily customized, if necessary, in order to individualize assessments as needed without unduly taxing stroke patients' limited tolerance for testing. Examiners did note that patients with significant expressive and receptive aphasias and hemiplegia/hemiparesis involving their dominant hands were often quite limited in their capacity to complete the battery, which heavily relies on verbal and written responses.

Finally, the protocol provided a sufficient* breadth* of useful data across cognitive domains, including cognitive efficiency, speech production, basic visuospatial/constructional skills, verbal learning and memory, and executive functioning. Although the* depth* of this data did not fully address all clinical issues for every patient, it significantly enhanced clinical understanding, thereby allowing neuropsychology providers to recommend other necessary services, including comprehensive neuropsychological assessments, behind-the-wheel driving evaluations, in-home safety evaluations, skilled nursing placements, and guardianship proceedings.

## 4. Conclusion

Poststroke cognitive impairment has been demonstrated to be a key predictor of patients' functional outcomes [8] and risks of disability and mortality [9] after stroke. Furthermore, systematic assessment of patients' cognitive capacities is necessary for using cognitive profiles in patient care due to the significant heterogeneity in poststroke cognitive outcomes [10, 11]. However, operational barriers often preclude the incorporation of neuropsychological services into routine poststroke care in many clinical settings.

In this large tertiary care center, the comprehensive stroke program and neuropsychology service collaboratively implemented a multidisciplinary stroke clinic to address the systemic obstacles that hindered effective, collaborative communication between stroke neurology and neuropsychology providers in their poststroke care efforts. As a result, multiple follow-up visits were condensed into one, reducing travel-related and other associated burdens on patients and reducing stroke patients' wait time between neurology and neuropsychology appointments by over 75%. Instead of requiring weeks or months for data transfer between providers, stroke neurology and neuropsychology providers were able to communicate directly regarding patient's cognitive outcomes in a single clinic appointment. After the first implementation year, the multidisciplinary clinic was associated with high satisfaction ratings from both providers and patients.

The half-hour neuropsychological assessment protocol for vascular cognitive impairment, recommended by the 2006 NINDS-CSN international harmonization standards for poststroke care [16], demonstrated good clinical feasibility and provided an efficient method for the provision of focused neuropsychological services in a clinical setting that often prohibits full, traditional cognitive assessments. In future studies, we hope to use the resulting data to provide benchmark evidence-based outcomes regarding the actual validity and reliability of the protocol itself and multidisciplinary stroke care overall. The current study, however, does not aim to address validity and reliability beyond the purpose of the NINDS-CSN's initial proposed recommendations. Given suggested clinical feasibility, additional studies are encouraged to explore validity and reliability of the protocols.

The multidisciplinary stroke clinic is a low-cost, easily implemented model of care that quickly becomes financially self-sustaining and is generalizable to both poststroke care teams in other hospitals and other healthcare groups that require multiple disciplines to provide patient care in a streamlined, efficient manner. Such significant increases in provider and patient satisfaction, as well as marked improvements in patient wait time and increases in available patient base, may be anticipated in other services that could utilize neuropsychological services on a regular basis, as well as associated disciplines such as physical therapy, occupational therapy, social work, and health psychology.

## Figures and Tables

**Table 1:** Multidisciplinary stroke clinic Press-Ganey patient satisfaction ratings (0 = lowest satisfaction, 5 = highest satisfaction), *N* = 16.

Satisfaction element	Mean	Standard deviation
Scheduling ease	4.87	0.35
Waiting room time	4.44	0.63
Degree to which you were informed about any delays	4.67	0.49
Friendliness/courtesy of the care provider	4.81	0.40
Explanations from care provider regarding your problem or condition	4.75	0.45
Concern care provider showed regarding your questions or worries	4.75	0.45
Care provider's effort to include you in decisions about your treatment	4.85	0.38
Instructions for follow-up care	4.43	0.65
Degree to which care provider talked with you using words you could understand	4.63	0.50
Amount of time care provider spent with you	4.80	0.41
Your confidence in this/these care provider(s)	4.69	0.48
Likelihood of your recommending this care provider to others	4.79	0.43
Convenience of our office hours	4.67	0.49
Our sensitivity to your needs	4.69	0.48
Our concern for your privacy	4.94	0.25
Ease of obtaining test results	4.54	0.66
Overall rating of care received during your visit	4.69	0.48
Likelihood of your recommending our clinic to others	4.69	0.48

**Table 2:** Multidisciplinary stroke clinic stroke neurology provider satisfaction ratings (0 = lowest satisfaction, 5 = highest satisfaction), *N* = 4.

Satisfaction element	Mean	Standard deviation
Effectiveness of scheduling before multidisciplinary stroke clinic	2.75	0.96
Effectiveness of scheduling after multidisciplinary stroke clinic	4.75	0.50
Data turnaround time before multidisciplinary stroke clinic	2.00	1.41
Data turnaround time after multidisciplinary stroke clinic	4.5	0.58
Perceived level of clinic value to patients	4.00	0.00
Perceived patient satisfaction with care model	4.25	0.50
Provider satisfaction with care model	4.5	0.58
Value multidisciplinary stroke clinic added to clinic practice	4.00	0.00
Impact of the multidisciplinary stroke clinic on comprehensive stroke service	4.75	0.50
